# Targeted Disruption of *py235ebp-1*: Invasion of Erythrocytes by *Plasmodium yoelii* Using an Alternative Py235 Erythrocyte Binding Protein

**DOI:** 10.1371/journal.ppat.1001288

**Published:** 2011-02-17

**Authors:** Solabomi A. Ogun, Rita Tewari, Thomas D. Otto, Steven A. Howell, Ellen Knuepfer, Deirdre A. Cunningham, Zhengyao Xu, Arnab Pain, Anthony A. Holder

**Affiliations:** 1 Division of Parasitology, MRC National Institute for Medical Research, London, United Kingdom; 2 Institute of Genetics, University of Nottingham, Nottingham, United Kingdom; 3 Parasite Genomics, Wellcome Trust Sanger Institute, Wellcome Trust Genome Campus, Hinxton, Cambridge, United Kingdom; 4 Protein Structure, MRC National Institute for Medical Research, London, United Kingdom; 5 Computational Bioscience Research Center, Chemical and Life Sciences and Engineering Division, King Abdullah University of Science and Technology, Thuwal, Saudi Arabia; Case Western Reserve University, United States of America

## Abstract

*Plasmodium yoelii* YM asexual blood stage parasites express multiple members of the *py235* gene family, part of the super-family of genes including those coding for *Plasmodium vivax* reticulocyte binding proteins and *Plasmodium falciparum* RH proteins. We previously identified a Py235 erythrocyte binding protein (Py235EBP-1, encoded by the PY01365 gene) that is recognized by protective mAb 25.77. Proteins recognized by a second protective mAb 25.37 have been identified by mass spectrometry and are encoded by two genes, PY01185 and PY05995/PY03534. We deleted the PY01365 gene and examined the phenotype. The expression of the members of the *py235* family in both the WT and gene deletion parasites was measured by quantitative RT-PCR and RNA-Seq. *py235ebp-1* expression was undetectable in the knockout parasite, but transcription of other members of the family was essentially unaffected. The knockout parasites continued to react with mAb 25.77; and the 25.77-binding proteins in these parasites were the PY01185 and PY05995/PY03534 products. The PY01185 product was also identified as erythrocyte binding. There was no clear change in erythrocyte invasion profile suggesting that the PY01185 gene product (designated PY235EBP-2) is able to fulfill the role of EBP-1 by serving as an invasion ligand although the molecular details of its interaction with erythrocytes have not been examined. The PY01365, PY01185, and PY05995/PY03534 genes are part of a distinct subset of the py235 family. In *P. falciparum,* the RH protein genes are under epigenetic control and expression correlates with binding to distinct erythrocyte receptors and specific invasion pathways, whereas in *P. yoelii* YM all the genes are expressed and deletion of one does not result in upregulation of another. We propose that simultaneous expression of multiple Py235 ligands enables invasion of a wide range of host erythrocytes even in the presence of antibodies to one or more of the proteins and that this functional redundancy at the protein level gives the parasite phenotypic plasticity in the absence of differences in gene expression.

## Author Summary

Malaria parasites invade erythrocytes where they develop and multiply before bursting out and invading fresh cells. There are sequential steps to invasion; early in the process, specific parasite proteins bind to molecules on the surface of the erythrocyte. Tight binding forms a junction between parasite and host cell leading to the next steps in the invasion process. Several of these parasite proteins, which establish contact with the host cell surface, are coded by gene families. One family, first described in the rodent parasite *Plasmodium yoelii* and found in all *Plasmodium* spp, is often referred to as the reticulocyte binding ligand family. In *P. yoelii* the proteins are called Py235 and are coded by at least eleven genes. Previously, we identified one family member which is the target of protective antibodies that prevent parasite invasion. Here we have deleted the gene for this protein and examined the consequences. Other members of the family take the place of the missing protein but their genes are not up-regulated. The family provides the parasite with the potential to recognize erythrocytes with different surface receptors and evade the binding of protective antibodies through plasticity at the level of its adhesion molecules.

## Introduction

Despite the recent renewed onslaught to tackle a disease that infects 300-660 million people and kills one million each year worldwide [1], the malaria parasite remains an elusive target. During the asexual blood stage, which is responsible for the disease, the parasite invades and develops within erythrocytes, but the precise molecular mechanisms employed to gain entry into the erythrocyte are still being worked out. A number of parasite adhesion proteins have been identified as important in the selection and invasion of host cells and have been grouped according to structural and sequence homology rather than host molecular specificity or cellular phenotype (reviewed in [2], [3], [4], [5]). The role of the actin-myosin motor complex in the invasion of erythrocytes is also being elucidated [6], [7]. Together, merozoite surface proteins, the adhesion ligands and the motor complex add up to a multifaceted molecular interaction that results in the successful selection and invasion of host cells [3], [4], [5]. Understanding the role played in the invasion cascade by adhesion proteins with homologues in both human and rodent *Plasmodium* is of paramount importance in the quest to design intervention tools that will inhibit invasion pathways and so kill the parasite and prevent disease.

Of the *Plasmodium* adhesion ligand families identified to date, one of the most studied is the erythrocyte binding ligand family (EBL), which includes *P. falciparum* erythrocyte binding antigen (EBA)-175 and the Duffy binding protein (DBP) of *P. vivax* and *P. knowlesi* (reviewed in [3], [4]) located in the apical organelles of the merozoite. A second group of high molecular mass adhesion proteins, which was first described in the rodent malaria parasite *Plasmodium yoelii* as Py235 [8], [9], is the reticulocyte binding-like (RBL) super family, so named because of sequence homology with the reticulocyte binding protein (RBP)-1 and RBP-2, of *Plasmodium vivax*. In *P. vivax,* these proteins are thought to be involved in erythrocyte selection as they bind to reticulocytes but not mature erythrocytes thereby restricting *P. vivax* to the invasion of reticulocytes [10]. *P. falciparum* contains a small group of genes coding for proteins with similarities to Py235 and PvRBP, the PfRH family [11], [12], [13]. In contrast to the PvRBP and PfRH gene families, which are small, the Py235 multigene family contains at least 11 members [9], [14], [15], [16], [17], [18]. Analysis of the sequences on fifteen contigs identified in the *P. yoelii* genome database [15], which represent members of the Py235 gene family (and some of which are incomplete), show they have overall conserved structural elements [16], [19], [20].

The Py235 proteins have been implicated in the selection, recognition and invasion of erythrocytes. For example, passive immunization of mice with monoclonal antibodies (mAbs) 25.77 and 25.37 specific for Py235, or immunization of mice with mAb 25.77-affinity purified protein restricts the growth of the virulent YM line of *P. yoelii* [8], [21]. In these experiments the invasion profile was switched from invasion of erythrocytes of all ages to invasion of only reticulocytes, suggesting that the antibodies prevent parasite recognition and invasion of mature erythrocytes. This restriction in cell specificity resulted in a non-lethal infection similar to that of the avirulent 17X line, in contrast to the normal lethal phenotype of the YM parasite. Of note is the finding that a combination of both protective mAbs together conferred greater protection [22], suggesting that the epitope recognized by each of the mAbs is not identical and may or may not be on distinct members of the family.

Py235 proteins are released in soluble form from parasitized cells maintained *in vitro*, and two of these are recognized by mAb 25.77 [23], [24], [25]. However, only one of these forms was detected binding specifically and preferentially to the surface of mature mouse erythrocytes [24]. Binding was to neuraminidase-resistant, chymotrypsin- and trypsin-sensitive erythrocyte receptors, and the binding was abolished by incubation with Py235-specific antibodies [25].

Several invasion pathways coexist in a single parasite as exemplified by *P. vivax* that requires selection of reticulocytes (using the RBPs [26]) that are Duffy blood group antigen positive, (using the DBP [27], [28]) to successfully gain entry into the host cell. In *P. falciparum* the Dd2 clone can switch from being dependent on sialic acid for entry into the erythrocyte, allowing it to invade neuraminidase-treated erythrocytes [29]. This change of phenotype has been found to be due to the up-regulation of PfRH4 [30], [31]. Polymorphism due to amino acid substitutions in the binding domain of PfRH5 leads to recognition of different erythrocyte surface receptors, [32]. That several pathways are available to a single parasite is further demonstrated by the observation that invasion into an enzyme-treated cell is not all or nothing even though enzymatic treatment goes to completion, (in *P. falciparum* [11], [33], and in *P. yoelii* [25]). Therefore, the invasion pathway of a parasite depends not only on the set of ligands expressed or silenced, some of which are coded by genes under epigenetic control [34], but also on a molecular hierarchy that determines which of the expressed ligands are used [2], [3]. This variant expression of adhesion-/invasion-related proteins is thought to be primarily driven by immune evasion although it may also help to increase the range of erythrocytes that can be invaded [35], [36].

Populations of *P. yoelii* asexual blood stage parasites express multiple members of the *py235* gene family [17], [36], [37]. Multiple gene products were detected in individual schizonts although only single products were identified in single merozoites, leading to the suggestion that the presence of the family allowed clonal phenotypic variation [38]. On the other hand, all merozoites within schizonts reacted with mAb 25.77 [17], suggesting that they either share the protein recognized by this antibody or the epitope is present on multiple members of the family. We have previously identified a specific erythrocyte binding member of the Py235 family (Py235EBP-1), which is recognized by mAb 25.77, and its corresponding gene *(py235ebp-1*[PY01365]*)* [20].

Here, we describe the effect on parasite growth *in vivo* of deleting the gene that encodes the Py235EBP-1 expressed in asexual blood stages of the virulent *P. yoelii* YM line in order to better understand the role of the Py235 protein family in erythrocyte recognition, binding, and merozoite invasion. We also examine the expression of other family members in this *py235ebp-1* knock out (KO) parasite line. Furthermore, we identify the proteins recognised by the other protective mAb 25.37 to obtain an understanding of the relationship in the invasion process between the two protective mAbs and the Py235 proteins they recognize. Whilst there is no difference in the level of expression of other genes in the family, other proteins compensate for the loss of the erythrocyte binding protein, highlighting the importance of functional redundancy to provide plasticity in interaction with the host.

## Results

### *py235ebp-1* (PY01365) can be deleted from the genome of the virulent *P. yoelii* YM line

**Figure 1:**
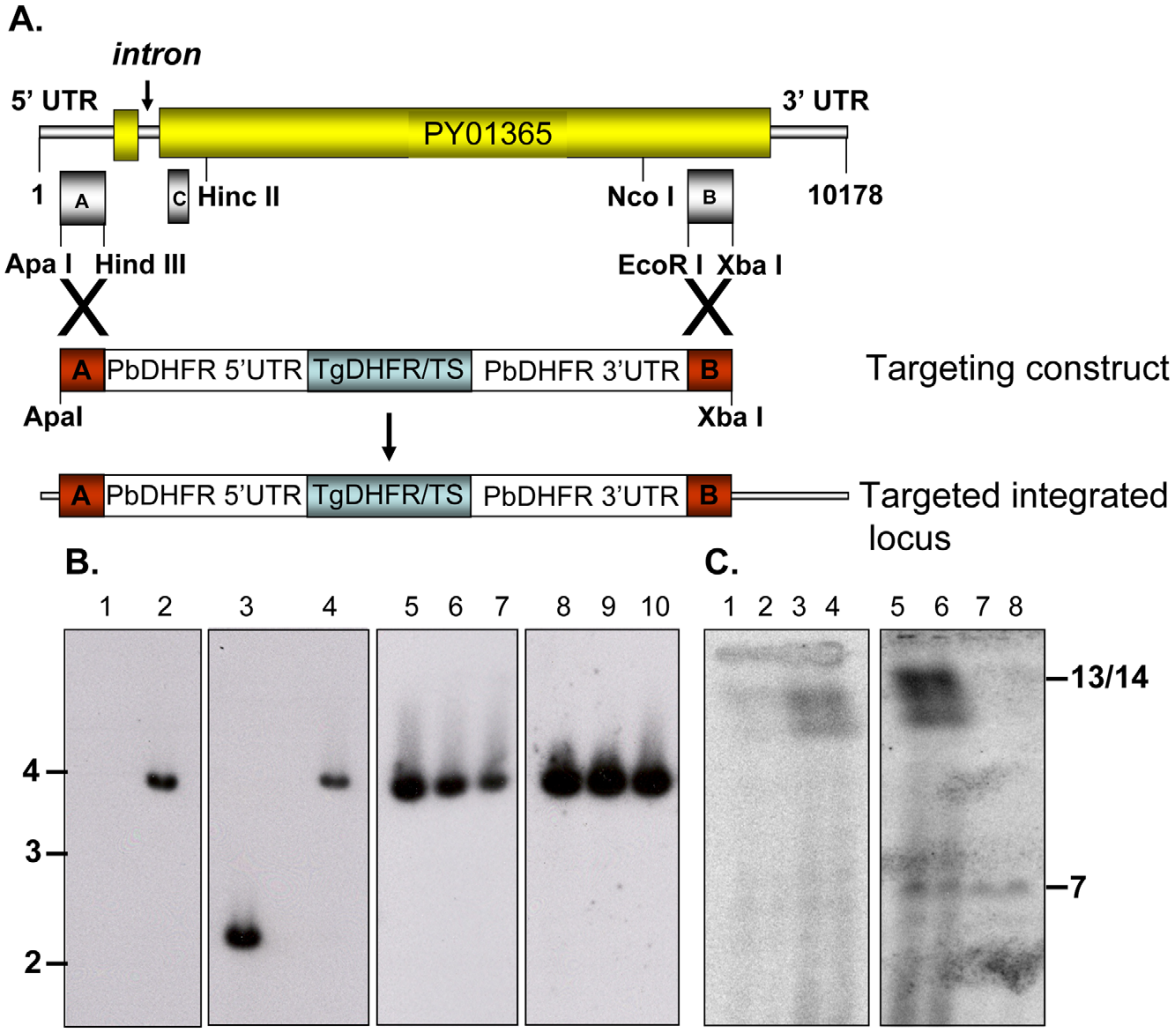
Targeted disruption of PY01365 (*py235ebp-1)* gene in *P. yoelii*. (A) Schematic showing the targeting construct used for gene disruption by double homologous recombination into the PY01365 gene and the resultant targeted integrated locus. The positions of the DNA probes (A, B and C) used in Southern blot analysis are indicated below the WT chromosomal locus. (B) Southern blot analysis of *P. yoelii* genomic DNA from WT, transgenic parasites and cloned transgenic parasites, digested with Pci1 and Acc651. Probes used are a 921bp fragment of the Tg/DHFR sequence (lanes 1, 2, 8, 9 and 10) and Fragment B, (lanes 3 to 7). The result of integration is seen at ∼3.8 Kb (lanes 2 and 4 to 10); the unmodified band at ∼2.3Kb present in WT DNA was detected with Fragment B probe alone (lane 3). (C). Chromosomes from a cloned transgenic parasite line (lanes 1, 2, 5 and 6) or WT parasites (lanes 3, 4, 7 and 8) were separated by pulse field gel electrophoresis and hybridized with Fragment C (lanes 1 to 4) and the Tg/DHFR probe (lanes 5 to 8). The position of chromosomes 13/14 and the endogenous DHFR on chromosome 7 is indicated.

Disruption of the *py235ebp-1* (PY01365) by insertion of the DHFR cassette by double homologous recombination (Figure 1A) was carried out. Southern blot analysis of digested gDNA from transfected parasites selected with pyrimethamine identified a single band of the expected size in this population, when the filter was probed with a fragment of DHFR/TS (Figure 1B, lane 2). In contrast, hybridization with the probe that binds to the 3′ coding region of *py235ebp-1* (Fragment B), detected DNA in both the wild type (WT) (2.3Kb) and the KO (3.8Kb) parasite lines as expected (Figure 1B, lanes 3 and 4). Four individual clones (1 to 3 shown) derived from the population of parasites gave a similar result with both fragment B (Figure 1B, lanes 5 to 7) and the DHFR/TS probe (Figure 1B, lanes 8 to 10), clearly showing that the PY01365 gene had been disrupted. Further evidence that the PY01365 gene had been deleted from the *P. yoelii* genome was obtained by chromosome analysis (Figure 1C). Hybridization with the probe that binds to a 5′ coding sequence of PY01365 (Fragment C), detected a signal only in the WT parasite lanes, (Figure 1C, lanes 3 and 4). Hybridization with a probe that binds the 3′ UTR of DHFR/TS detected both the modified *py235ebp-1* locus (chromosome 13/14) in the KO parasite line (Figure 1C, lanes 5 and 6) and the endogenous *dhfr* locus (chromosome 7) in both KO and WT parasites (Figure 1C, lanes 5 to 8). Hybridization of a chromosome blot with the Fragment B probe identified a band in all the lanes as expected (data not shown).

### *py235ebp-1* (PY01365) is a single copy gene in the *P. yoelii* YM genome

**Figure 2:**
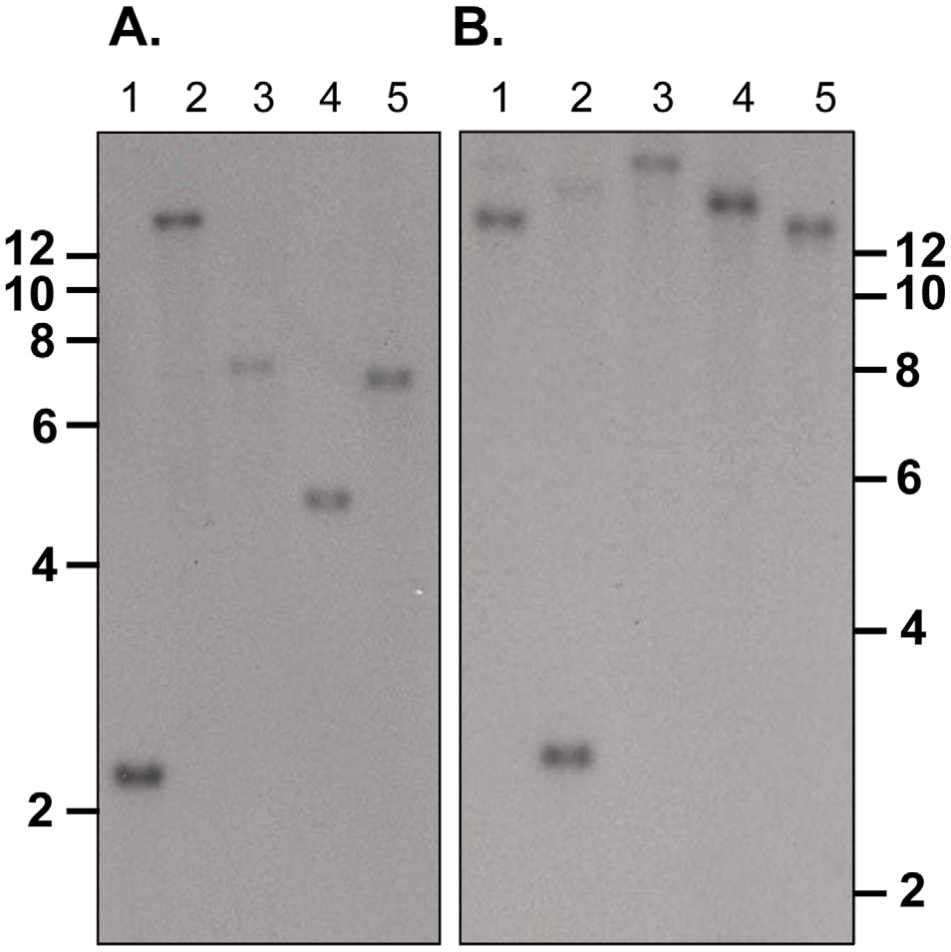
Determination of the number of copies of PY01365 in the genome. Southern blot analysis of WT genomic DNA digested with restriction enzymes. Panel A probed with Fragment C and digested with Hinc II and Hind III (lane 1), Hinc II and Acc 651 (lane 2), Hinc II and Bgl II (lane 3), Hinc II and Pvu I (lane 4) and Hinc II and Sca I (lane 5). Panel B probed with Fragment B and digested with Nco I and Sca I (lane 1), Nco I and Pvu I (lane 2), Nco I and Pst I (lane 3), Nco I and Bgl II (lane 4) and Nco I and Acc 651 (lane 5). The migration of size markers (Kb) is indicated.

Southern blot analysis of ten sets of double restriction enzyme digested gDNA from WT *P. yoelii* YM-parasitized erythrocytes probed with either Fragment B or C gave single bands under low stringency washes (Figure 2). These data suggest that PY01365 is a single copy gene in the line of *P. yoelii* YM parasites used in this study. This conclusion is also supported by the absence of any RNA-Seq reads mapping to any part of PY01365 from the PY01365-KO parasite.

### Merozoites in both WT and PY01365-KO parasitized erythrocytes express proteins with epitopes recognized by both mAb 25.77 and 25.37

**Figure 3:**
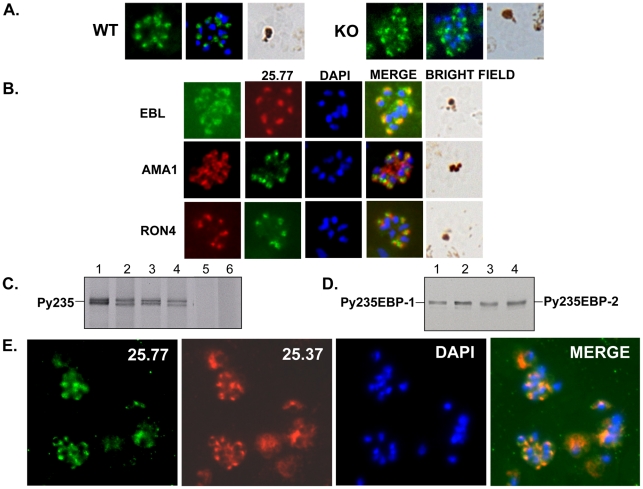
Detection of Py235 protein expression. (A) Indirect fluorescent antibody assay using mAb25.77 on 1% formaldehyde-fixed thin blood smears of WT or PY01365-KO schizonts. For each parasite line the first panel shows mAb 25.77 reactivity, the second shows mAb 25.77 reactivity and parasite nuclei labelled with DAPI, and the third shows the bright field image. (B) a dual labeling experiment of WT parasitized erythrocytes. In the first column of panels the antibodies used were rabbit anti-EBL, mAb 45B1 (AMA1) and mAb 48F8 (RON4), each of which was detected by the appropriate fluorescence-labelled second antibody. In the second column of panels the same cells were stained with mAb 25.77, which was detected either with a fluorescence-labelled antibody (red) or was directly conjugated to a fluorescent dye (green). The third column of panels shows parasite nuclei labelled with DAPI, with the fourth column of panels showing the overlay of all three previous panels in the row (MERGE). The fifth column of panels shows the bright field images. (C) Immunoprecipitation analysis of WT (lanes 1, 3 and 5) and PY01365-KO (lanes 2, 4 and 6) parasite soluble proteins released into culture supernatant using mAbs 25.77 (lanes 1 and 2), 25.37 (lanes 3 and 4) and normal mouse serum (lanes 5 and 6). The Py235 proteins detected by this analysis are shown. (D) Erythrocyte binding assay: detection of a single protein band eluted from erythrocytes and immunoprecipitated using mAbs 25.77 (lanes 1 and 2) and 25.37 (lanes 3 and 4) and derived from WT (lanes 1 and 3) and KO (lanes 2 and 4) parasites, respectively. The proposed identities (Py235EBP-1 and Py235EBP-2) are indicated. (E) Alexa Fluor 488-conjugated 25.77 (green) and Alexa Fluor 594-conjugated 25.37 (red) were used in a dual labeling experiment of WT parasitized erythrocytes. The third of four panels of an identical field shows parasite nuclei labelled with DAPI, with the fourth panel showing the overlay of all three previous panels (MERGE). Py235 proteins are recognised by both mAbs in each schizont.

The mAb 25.77 had previously been used to identify Py235EBP-1, the product of the PY01365 gene. By IFA, this mAb gives a punctuate pattern of fluorescence in the WT parasite line (Figure 3A). Surprisingly, a similar pattern was also observed for the PY01365-KO parasite line, even though Py235ebp-1 is no longer being expressed. The pattern of reactivity (Figure 3B) was similar but not identical to that of antibodies specific for the micronemal protein, Apical Membrane Antigen 1 (AMA1) [39], the erythrocyte binding ligand protein (EBL), which has a dense granule location in this parasite line [40], and rhoptry neck protein 4 (RON 4) [41]. Furthermore, when proteins released into *in vitro* culture supernatant from radiolabeled WT and PY01365-KO parasitized erythrocytes were immunoprecipitated using mAbs 25.77 and 25.37 (Figure 3C), or bound to erythrocytes, eluted and then immunoprecipitated (Figure 3D), both mAbs recognized proteins of approximately 235 kDa showing that Py235 proteins were expressed by both WT and PY01365-KO parasite lines. Clearly the Py235 proteins now expressed by the KO parasite line, although at least in part different to those being expressed by the WT parasite, share common epitopes bound by the antibodies.

Further confirmation that merozoites express epitopes recognized by both mAb 25.77 and 25.37 was obtained using WT parasitized erythrocytes in a dual labeling fluorescent assay. Alexa Fluor 488-conjugated mAb 25.77 and Alexa Fluor 594-conjugated mAb 25.37 were used to probe the same thin blood smears of mixed stage WT *P. yoelii* YM parasites (Figure 3E). Overlay of the individual images showed clearly that both antibodies recognized the same parasites.

### Two genes comprised of three different contigs encode the Py235 proteins recognized by mAb 25.37

**Figure 4:**
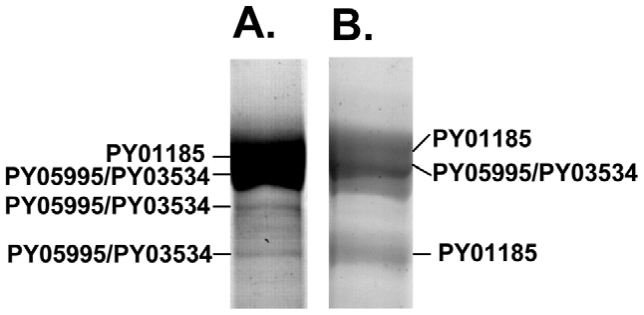
Identification by mass spectrometry analysis of proteins recognized by mAb 25.37 in extracts of schizonts and culture supernatants. Py235 proteins were purified from detergent-lysed erythrocytes containing WT parasites and from the supernatant of cultured parasites by affinity chromatography on mAb 25.37 and resolved by SDS-PAGE on a 5% polyacrylamide gel. Protein bands were stained, excised, reduced and alkylated and digested with trypsin and then the peptides were analyzed by MALDI-ToF mass spectrometry. The peptide mass fingerprints obtained identified two Py235 proteins in schizont extracts (PY01185 and PY03534, panel A),with the products of an additional contig (PY05995) also in the supernatant, (panel B).

**Figure 5:**
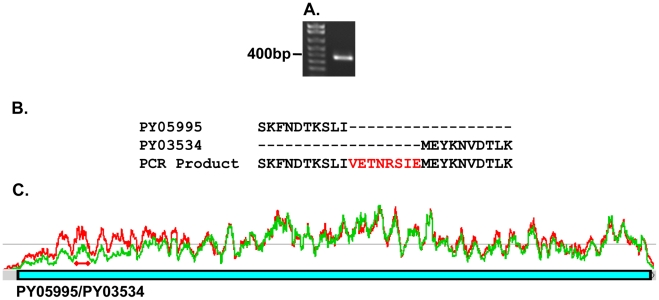
PCR amplification of a py235 gene fragment spanning two contigs, PY05995 and PY03534. (A) A single PCR product of 392bp was obtained using a forward primer designed on DNA sequences from within the last 200 nucleotides of the 3′ coding sequence of PY05995 and a reverse primer from within the first 200 nucleotides of the 5′ coding sequence of PY03534. (B) Alignment of part of the amino acid sequence translated from the directly sequenced PCR product with the relevant PY05995 and PY03534 amino acid sequences (obtained from the NCBI database). The PCR product sequence aligned perfectly with that of the two contigs and filled a 24 nucleotide gap coding for the eight amino acid residues indicated in red. (C) Confirmation of PY05995 and PY03534 concatenation by RNA-Seq. The two sequences were linked in the RNA-Seq data by 153 read pairs that mapped to the end of PY05995, and the beginning of PY03534. The graphs present the expression in the WT (green) and PY01365-KO (red) parasite lines. The two red diamonds joined by a line indicates the position of the qPCR primers.

The gene encoding the protein recognized by mAb 25.77 and expressed in WT parasites has previously been identified. A second protective mAb 25.37 also recognizes Py235 proteins and we wished to identify the protein(s) to which it binds. To identify the corresponding genes, peptide mass fingerprinting was carried out on the Py235 proteins affinity purified using mAb 25.37 from both schizonts (Figure 4A) and from culture supernatant of WT parasites maintained *in vitro* (Figure 4B) and fractionated by SDS-PAGE on a 5% gel. The peptides detected were derived from three contigs in the genome database; one contained a full length (8172bp) py235 gene sequence, PY01185, and the remaining two contained partial gene sequences (Table 1). Contig PY05995 is 2685bp in length and contains sequence that aligned with the 5′ end of other Py235 gene family members, while PY03534 (5478bp) aligned with the 3′ of other Py235 genes. To establish whether or not these two contigs are part of the same gene, primers designed to the 3′-sequence of PY05995 and to the 5′-sequence of PY03534 were used to amplify sequence from gDNA, and gave a single PCR product of the expected size, 392bp (Figure 5A). Sequence analysis and alignment with the gene sequences from the database showed perfect alignment (Figure 5B) and confirmed that the contigs were part of the same single full length Py235 gene, PY05995/PY03534. This conclusion was further confirmed by read pairs of the RNA-Seq data. 153 mates mapped to the end of PY03534 and the beginning of PY05995, and the entire gene could be assembled from the mapping reads (Figure 5C).

**Table 1:** Genes identified by mass spectrometry analysis of proteins purified from WT and KO parasites.

mAb	Parasite preparation	WT	Py01365-KO
25.77	Schizont	PY01365 (145)	Py01185 (157)
			PY05995/PY03534 (136)
	Culture supernatant	Py01365 (93)	Py01185 (98)
			PY05995/PY03534 (46)
25.37	Schizont	PY01185 (98)	ND
		PY05995/PY03534 (90)	
	Culture supernatant	PY01185 (40)	ND
		PY05995/PY03534 (90)	

All reported MASCOT scores (in brackets) are significant at a minimum of p<0.005; ND, not done.

### Comparison of the sequences of the proteins bound by mAbs 25.37 and 25.77

Sequence data for the Py235 family was aligned and examined for structural features. Partial or full length sequences were compiled from the literature [15], [16], [17], [19], [42], [43] resulting in eleven essentially full length protein sequences, one almost full length but lacking the C-terminus and two others, one representing the N- and the other the C-terminal sequence of one or two further genes. The PY01365, PY01185, and PY05995/PY03534 sequences form a discrete subset of the family with a degree of similarity in pairwise alignment of greater than 80% at the amino acid sequence level (Table 2). None has the Asp-Ile-Asn (DIN) repeats close to the C-terminus of the protein found in some members of the family. A further subgroup contains genes 11, 10, PY03184_E3, PY02104_E5, and PY04438_PY0618_E8.

**Table 2:** Protein sequence identity and similarity.

	Protein sequence identity/similarity (%)								
Gene product	PY01185	PY05995/PY03534	PY00649	PY04630	11	PY03184_E3	10	E8	E5_PY2104**
**PY01365**	68/81	68/80	57/75	57/74	50/68	51/69	50/68	51/70	50/69
**PY01185**		86/93	60/76	59/75	51/69	51/70	50/69	51/70	52/70
**PY05995/PY03534** *			59/75	58/75	51/69	52/70	49/68	51/69	51/69
**PY00649 (E1)** **				85/91	63/76	64/77	63/77	63/78	63/77
**PY04630 (E2)** **					63/76	64/77	60/75	60/76	60/75
**11 (PY03184)** **						98/99	73/85	73/83	70/83
**PY03184_E3** *							73/85	73/84	70/83
**10 (PY04960)** **								77/87	72/84
**E8 (PY06018)** **									72/84

Pairwise alignment was carried out using published sequences.

*Indicates where a gene has been compiled by the alignment of more than one contig.

**Indicates where a contig or partial sequence (named in brackets) is a smaller fragment with identical sequence within the region of overlap.

### Expression of proteins encoded by the Py235 gene family in the PY01365-KO parasite and their recognition by mAb 25.77

**Figure 6:**
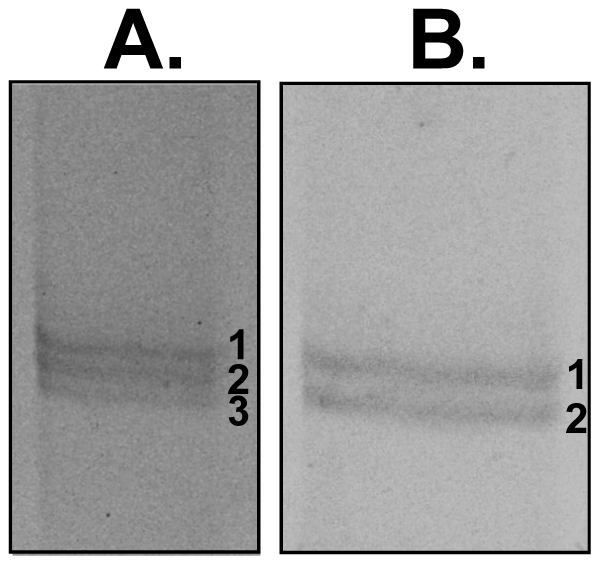
Identification using mass spectrometry analysis of proteins from the PY01365-KO parasite line, which are recognized by mAb 25.77. Py235 proteins were purified from a detergent-lysate of PY01365-KO parasitized erythrocytes or the corresponding culture supernatant by affinity chromatography on mAb 25.77. Fractions of purified protein eluted sequentially from the affinity column were resolved by SDS-PAGE on a 5% polyacrylamide gel. The protein bands were excised, reduced and alkylated and digested with trypsin, and the peptides analyzed by MALDI-ToF mass spectrometry. The peptide mass fingerprints were used to identify the genes encoded by the purified proteins. (A) Proteins purified from schizonts: band1, PY01185; band 2, PY01185; band 3, PY03534. (B) Proteins purified from culture supernatant: Band 1, PY01185; band 2, PY03534. Essentially two Py235 contigs were identified from both the schizont and supernatant preparations.

We were interested to identify the Py235 proteins expressed by the PY01365-KO parasite line that were recognised by mAb 25.77 in the absence of Py235EBP-1. Py235 proteins affinity purified using mAb 25.77 from both detergent-solubilised parasite preparations (Figure 6A) and culture supernatant (Figure 6B) from the PY01365-KO parasite line were fractionated on a 5% SDS-PAGE gel and processed for mass spectrometry analysis. MASCOT searches using the peptides and the NCBI database gave significant matches to three Py235 contigs, PY01185, PY05995 and PY03534 (Table 2), mirroring the results obtained with mAb 25.37 and WT parasites. The results suggest that mAb 25.77 has a higher affinity for PY01365, than PY01185 and PY05995/PY03534 protein products, since in the absence of PY01365 mAb 25.77 was able now to detect the other proteins. While mass spectrometry analysis of proteins affinity purified from WT parasites using mAb 25.77 routinely clearly identified PY01365 (Py235EBP-1) (Table 2), peptides derived from PY01185, PY05995 and PY03534 were also present in small amounts (data not shown). Due to the high number of unique peptides required for positive identification of these large proteins, the few unique peptides obtained for PY01185, PY05995 and PY03534 was insufficient.

### Identification of a second Py235 protein that binds to the erythrocyte surface and is recognized by mAb 25.37 in extracts of WT parasites and by mAb 25.77 in extracts of the PY01365-KO parasite line

Radiolabeled proteins from WT and PY01365-KO parasites that had been released into the supernatant of *in vitro* cultures were used in erythrocyte binding assays. Proteins bound to and eluted from the erythrocyte surface were immunoprecipitated using mAbs 25.77 and 25.37. These mAbs recognized single protein bands of approximately 235 kDa in this fraction (Figure 3C and D). We have shown previously that of the several biosynthetically-labeled Py235 proteins released into the supernatant of parasites incubated *in vitro*, only one binds to the surface of erythrocytes and is recognized by mAb 25.77 (Py235EBP-1). Similarly, we now show that mAb 25.37 also recognizes two Py235 proteins released into culture supernatants (Figure 3C) and that only one of them, the upper of the two bands (Figure 3D), binds to erythrocytes. This upper band has been identified as the protein encoded by the gene, PY01185 (Figure 4A and B), identifying another Py235 protein that binds to the surface of erythrocytes and is recognized by the protective mAb 25.37. This protein has been designated Py235 erythrocyte binding protein-2 (Py235EBP-2), as the second known erythrocyte binding protein from this family and encoded by the PY01185 gene. Our result suggests that in WT parasites there are at least two erythrocyte binding proteins, Py235EBP-1, encoded by PY01365 and Py235EBP-2 encoded by PY01185. In the PY01365-KO parasite line, the erythrocyte binding protein is Py235EBP-2.

By western blotting, similar amounts of Py235 protein were detected by mAbs 25.37 and 25.77 in extracts of both WT and PY01365-KO parasites (Figure S1) indicating that there has been no compensatory change in protein levels such as upregulation of Py235EBP-2.

### Expression of Py235 genes in the WT and PY01365-KO parasite lines

To test the hypothesis that there had been a switch, for example the up-regulation of other members of the Py235 family expressed in the PY01365-KO parasite line, two methods were used: quantitative RT-PCR (qPCR) for some specific members and RNA-Seq for all known members of the family.

**Figure 7:**
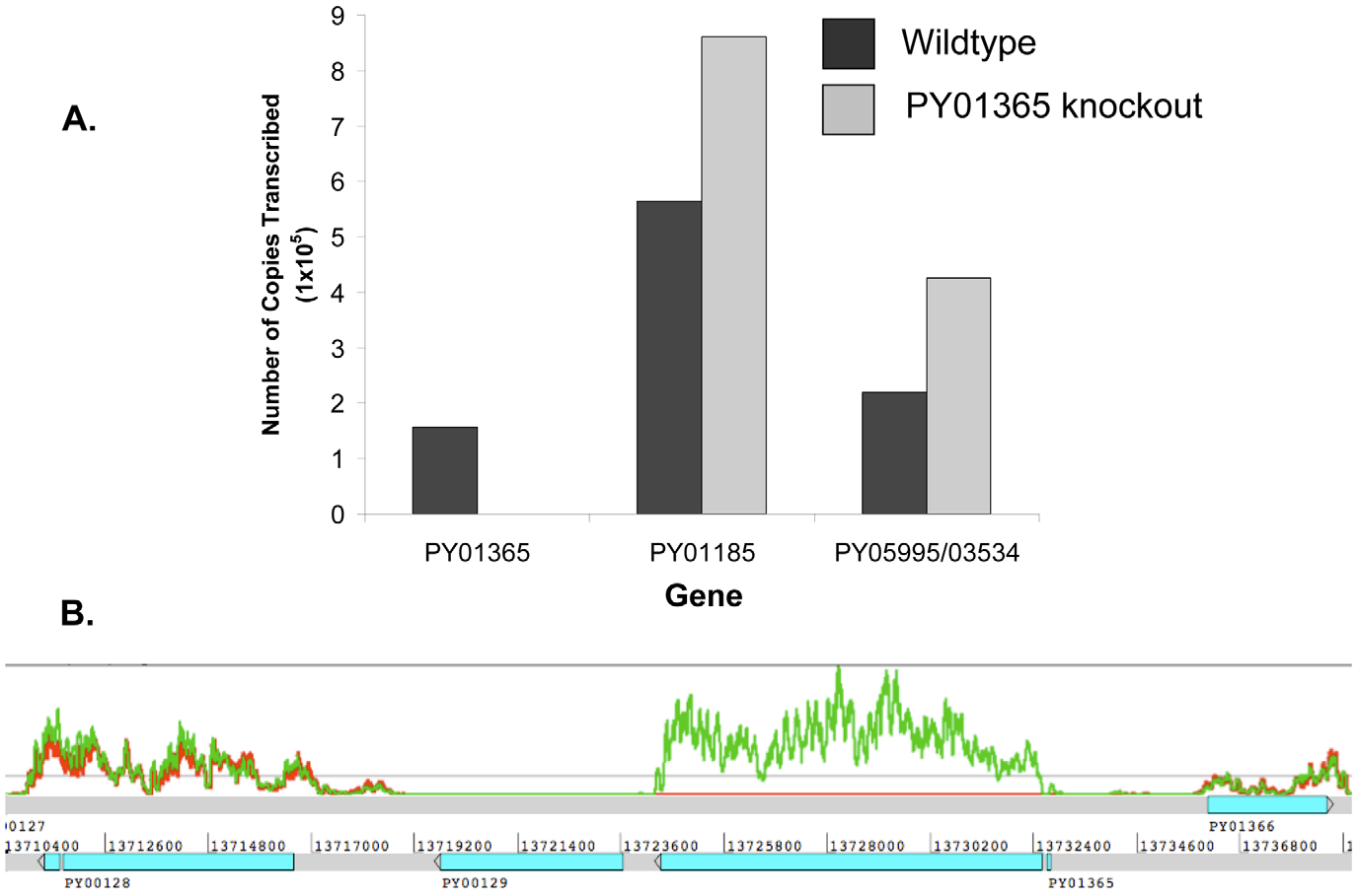
Analysis of Py235 gene family expression. (A) Quantitative real time RT-PCR (qPCR) analysis of genes that encode proteins recognized by the protective mAbs. Comparison of transcription levels of genes, expressed as number of copies transcribed per 1×10^−9^ g DNA. qPCR was carried according to the MIQE guidelines. All qPCR reactions were set up in triplicates. The result of a representative experiment is shown. Transcripts from three PY235genes: PY01365, PY01185 and PY05995/PY03534 were examined from both WT and PY01365-KO parasites. (B) RNA-Seq coverage plot for PY01365 and flanking genes. The gene models are in light blue. The lines represent the coverage plots for the numbers of reads: the green line is for WT and the red line for PY01365-KO parasite lines, respectively. The knocked out gene PY01365 is not expressed, unlike two neighboring genes, PY00128 and PY01366 that are expressed at similar levels in both parasite lines. PY00129 is not expressed.

qPCR was carried out using primers specific to the genes of interest and to reference genes coding for PyEBL (PY04764), which is expressed at the same developmental stage as Py235 proteins, and the gene for the constitutively expressed protein Pyβ-tubulin (PY05711) (Table 2). Of the 3 genes in the Py235 family that were examined, PY01365 had the lowest transcription level followed by PY05995/PY03534, with PY01185 having the highest transcription level in the WT parasite line (Figure 7a).

Two reference genes, PyEBL and Pyβ-tubulin had similar amounts relative to each other in both parasite lines. The quantification cycle (Cq) value obtained for PY01365 was similar to those for the negative controls (-RT or no template control), confirming that PY01365 had been deleted from the genome of *P. yoelii* YM in the PY01365-KO line. Fold change transcriptional calculations between the KO and WT lines were made. There was a fold change increase of 1.5 in the transcription level of PY01185 in the KO line, and for PY05995/PY03534 the fold change increase was 1.9. The measured fold change decrease of PY01365 in the KO line was 350, showing its absence in the PY01365-KO line.

In the RNA-Seq data, the Pearson correlation of all expressed genes between both parasite lines is nearly 0.99 (0.9899136), taking the log of the geometric mean [44] (Figure S2). Also the ratio of expression between the two reference genes of the qPCR (PY04764 and PY05711) was between 0.9 and 1.1 (Table 3). The data show that within the family, in the WT parasite, two genes are very poorly expressed (PY02104 and PY06381). Preliminary analysis of *P. yoelii* YM genomic DNA suggests that PY06381 is absent from this genome (data not shown). Of the remaining genes all are expressed (geometric means 159.56 to 725.4) with the PY05054 transcript being most abundant. Comparing the WT and PY01365-KO lines, as expected PY01365 is not expressed in the PY01365-KO parasite line but is clearly present and expressed in the WT, confirming the deletion of the PY01365 gene (Figure 7b). For the other members of the family the average ratio of expression in KO versus WT lines was 1.022 and for PY05995 and PY01185 it was 1.406 and 0.999, respectively. This indicates that there was no compensatory significant upregulation of expression of any of the other Py235 genes in the KO parasite.

**Table 3:** Expression of members of the Py235 family and two control genes determined by RNA-Seq.

GeneID	PY01365-KO geomean	WTgeomean	KO/WTratio
PY05995	224.35	159.56	1.41
PY04438	375.55	290.44	1.29
PY05995/PY03534	285.07	242.87	1.17
PY03534	296.67	280.52	1.06
PY06018	492.73	470.69	1.05
PY05054	739.29	725.4	1.02
PY01185	345.7	346.1	1.00
PY02104	9.85	9.9	0.99
PY04930	278.96	280.66	0.99
PY06381	1	1.01	0.99
PY00649	189.27	194.45	0.97
PY03184	397.21	424.47	0.94
PY02033	255.2	278.54	0.92
PY04630	225.25	255.74	0.88
PY03432	281.15	347.29	0.81
PY01365	1.12	398.3	0.00
PY04764*	301.64	328.15	0.92
PY05711*	1947.33	1772.46	1.10

The expression is the geometric mean of the mapping RNA-Seq reads for each parasite line. * erythrocyte binding protein (PY04764) and β-tubulin (PY05711).

### Similar *in vivo* growth kinetics are observed for both WT and PY01365-KO parasite lines

**Figure 8:**
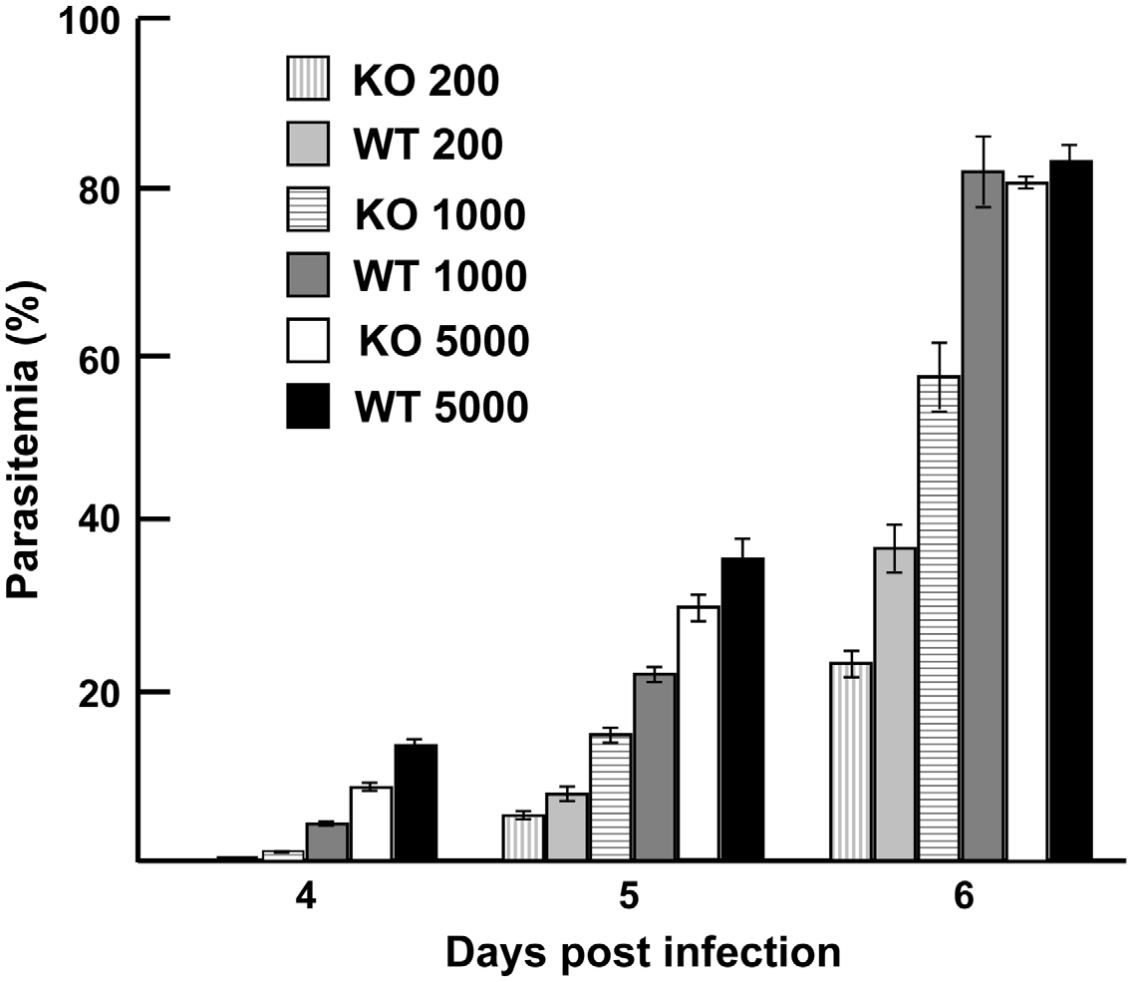
Parasite growth *in vivo.* The course of parasite growth was monitored in groups of 5 Balb/c mice infected intravenously (i.v.) on day 0 with 200, 1000, or 5000 WT or PY01365-KO parasitized erythrocytes. A Giemsa-stained blood smear from each mouse was made daily from day 3, the parasitaemia was counted and used to monitor the course of infection up to day 6. Error bars are standard error of mean (SEM). The bars are: 200 KO (vertical patterned), 200 WT (light gray), 1000 KO (horizontal patterned), 1000 WT (dark gray), 5000 KO (white), and 5000 WT (black).

To evaluate the phenotypic effect of deleting *py235ebp-1* from the genome of the virulent *P. yoelii* YM line, for example on the age of the host cell invaded or on the course of infection, groups of 5 mice were injected with parasitized erythrocytes. *P. yoelii* parasites of the KO line showed no changed preference for a particular host cell type relative to WT parasites. All host cell types, both mature erythrocytes and reticulocytes, were invaded, indicating that deletion of the PY01365 gene (*py235ebp-1*) did not restrict the parasites to invasion of reticulocytes. In mice made reticulocytemic there was no difference in cell preference or growth rate between the two parasite lines (data not shown). Parasite growth kinetics for all groups of mice were very similar and there was no clear difference in the parasite multiplication rate (Figure 8). Only in the group injected with a thousand parasitized erythrocytes was there a significant reduction in parasite growth, when comparing the KO and WT parasite lines (P<0.05). Disruption of the PY01365 gene was not lethal to the parasite and no phenotype was detectable with respect to the age of host cell invaded.

## Discussion

We were interested to examine the effect on growth *in vivo* of deleting the gene that encodes the Py235EBP-1 protein expressed in asexual blood stages of the virulent *P. yoelii* YM line. Additionally, we wished to examine the expression of other family members in this *py235ebp-1-*KO parasite line and identify the proteins recognised by the other protective mAb 25.37 in WT parasites.

We have previously shown that although there are several biosynthetically-labeled soluble Py235 proteins released into the supernatant of parasites incubated *in vitro*, only one of these proteins binds to the surface of erythrocytes and is recognized by the protective mAb 25.77 [24]. We show here that the second protective mAb 25.37 also recognizes two Py235 proteins in the *in vitro* culture supernatant, namely the products of PY01185 and PY05995/PY03534. We have obtained peptide mass and sequence information from the Py235 proteins either purified from parasitized erythrocytes or from the in vitro culture supernatant, which identifies the corresponding genes as members of the Py235 family. As in the case of the proteins recognized by mAb 25.77, only one of the two Py235 proteins, the upper of the two protein bands, PY01185, binds to erythrocytes. Therefore PY01185 is the gene identified as coding for the erythrocyte binding protein recognized by 25.37, which has been designated Py235 erythrocyte binding protein-2 (Py235EBP-2).

We sought to identify the proteins being expressed by the *py235ebp1*-KO parasite line that could still be recognized by mAb 25.77. Interestingly, proteins affinity purified from both detergent solubilized parasites and culture supernatant using mAb 25.77 were shown to be Py235EBP-2 (PY01185) and PY05995/PY03534 - the same gene products recognized by mAb 25.37 in WT parasites. Our results clearly show that in WT parasites there are two erythrocyte binding proteins, namely, Py235EBP-1, recognized by the protective mAb 25.77 and encoded by PY01365 and Py235EBP-2 recognized by the protective mAb 25.37 and encoded by PY01185. In the PY01365-KO parasite line, the erythrocyte binding protein recognized by mAb 25.77 is Py235EBP-2. Immunofluorescence studies indicate that each merozoite within a schizont expresses proteins recognized by both protective mAb 25.77 and 25.37 and that proteins recognized by the protective mAbs are expressed by all merozoites; this confirms and extends the conclusions of Narum et al [17]. The location of the proteins still needs to be resolved: in the immunofluorescence studies there was only partial overlap of mAb 25.77 staining and that of other antibodies specific for microneme, dense granule and rhoptry neck proteins.

It has been reported that some *P. yoelii* lines contain two copies of the PY01365 gene [45]. For the YM line we have analysed, the data indicate that only one copy of the gene is present. This conclusion is based on the Southern blot analysis of separated chromosomes, and of digested gDNA, and is supported by the qPCR and RNA-Seq analyses. However, other members of the gene family with a significant homology to PY01365 may be detected with certain probes at low stringency. This is in agreement with work carried out by Iyer et al [36]. The absence of the PY06381 gene in the YM genome is consistent with an extra gene being detected on chromosome blots of 17X parasites [18].

We examined gene transcription in both the WT and PY01365 parasites by qPCR and as expected, there was no difference in the mRNA levels of PyEBP and Pyβ-tubulin between the WT and KO parasite lines. Of the 3 genes in the *py235* family whose transcriptional level was examined, PY01365 had the lowest transcription level followed by PY05995/PY03534 and then PY01185. This result is in contrast to that of Iyer et al [36] who reported that PY01365 was the most highly expressed Py235 family member. This difference may be due to the use of different *P. yoelii* YM parasite lines with different gene copy numbers. There was a small difference in the level of transcription of PY01185 and PY05995/PY03534 between the WT and KO parasite lines. A more detailed analysis of the Py235 family using RNA-Seq indicated that most of the Py235 gene family is transcribed in these asexual blood stage parasites and there is no change following the deletion of PY01365 other than in the absence of products from this gene. Thus the redundancy in the function of this family must occur at the protein level rather than being reliant on genes being up-regulated; PY01185 protein probably takes over the function of PY01365, although the level and role of other Py235 proteins cannot be addressed. This result is in contrast to the picture in *P. falciparum* where individual RH protein genes are under epigenetic control and change in expression can lead to change in receptor recognition and host cell invasion pathway. It is possible that the expression of most of the Py235 genes at the same time could contribute to the noted virulence of the YM parasite [46].

Targeted disruption of *py235ebp-1* (PY01365) did not lead to a change in the invasion phenotype. Although there was a significant difference in the course of infection in groups of mice injected with 1000 parasitized erythrocytes (P<0.05), there was no significant difference in the groups of mice that received either 200 or 5000 WT and KO parasites. It will be of interest to delete both PY01365 and PY01185, since this double deletion might be expected to result in a much more severe phenotype.

The most puzzling result was that we were unable to detect at a significant level by affinity purification the PY01185 and PY05995/PY03534 proteins in extracts of WT parasites using mAb 25.77 even though the transcripts were present in the parasite at similar or higher levels than that of PY01365. In contrast these proteins were clearly detectable with mAb 25.77 in extracts from the KO parasite line using exactly the same methodology and could be purified from the extract of WT parasites using the second mAb, 25.37. One limitation of MALDI-TOF fingerprinting is that large proteins require a relatively large number of matched peptides to generate a significant MASCOT score. The few peptides unique to PY05995/PY03534 identified on detailed analysis of the peptide mass finger print data were insufficient to establish the presence of PY01185 and PY05995/PY03534 in the proteins extracted from WT parasite using mAb 25.77 and so these proteins were below the level of detection by mass spectrometry. Even if the level of transcription as judged by qPCR and RNA-Seq did not correlate with the level of protein expression, this does not explain the discordant results obtained with the two antibodies. It is possible that the two mAbs may recognize common binding domains in the Py235 proteins but with different affinities, dependent upon the precise amino acid sequence of the antigens. It is conceivable that in the absence of Py235EBP-1 (which may have a higher affinity for mAb 25.77) other members of the family, such as Py235EBP-2, and PY05995/PY03534, can now bind to mAb 25.77.

In the absence of the gene coding for the Py235EBP-1, other family member proteins carry out the same function; this redundancy facilitates binding and erythrocyte invasion. We show that the removal of the erythrocyte ligand expressed by PY01365, in the *py235ebp1-*KO line allows other expressed members of the py235 gene family to be used. In this instance, there was no change in phenotype with respect to the age of host cell invaded and mediated by the new set of parasite ligands. The invasion phenotype/pathway of a parasite depends not only on the set of ligands expressed or silenced, but also on a molecular/functional hierarchy that determines which of the expressed ligands are used [3], reviewed by Cortes [2]. Several pathways probably coexist in a single parasite so that invasion into different cells such as those in different mammalian hosts or experimentally generated, such as enzyme-treated cells is not all or nothing even though enzymatic treatment goes to completion, (in *P. falciparum*, [11], [33] and in *P. yoelii* [25]). Our data would also fit in with the ‘limited space hypothesis’ proposed for the *P. falciparum* (PfRH family) whereby the position of a particular PfRH ligand at the apex of the merozoite determines which ligand is used for invasion [47], [48]. In this current study, perhaps the absence of Py235EBP-1 allows space for the binding of another member of the Py235 family member, such as Py235EBP-2 (PY01185) to initiate erythrocyte invasion.

Different levels of protein expression in *Plasmodium* that are not matched by the level of transcription as seen by qPCR or RNA-Seq may arise through post-transcriptional controls of these proteins [34]. The sub-telomeric location of invasion-associated multigene families, including the Py235 family [18], may be important for variant expression, alternatively, new forms of protein regulation at the level of translation [49] may occur. However, none of these mechanisms appears to contribute to our findings because the level of proteins seems to be unchanged.

Analysis of the protein sequences we have identified showed that they are coded by a subset of the Py235 family. For example, they all lack the short repetitive sequence based on the tripeptide, Asp-Ile-Asn (DIN), which is located just N-terminal to the transmembrane domain. The significance of this is obscure but the observation does cast doubt on the validity of using this repeat sequence as a diagnostic marker for the expression of all genes in the Py235 family [37], [38], [50].

In this study we have shown by targeted disruption that the *py235ebp-1* (PY01365) gene is not essential to the parasite and the KO did not result in a change in the invasion phenotype with respect to the age of mouse cell invaded or the parasite growth rate. However, deletion of *py235ebp-1* did seem to result in an alteration in the level or accessibility of other Py235 protein family members such that they became able to bind to mAb 25.77; the proteins coded by PY01185 and PY05995/PY03534 appeared to compensate for the absence of *py235ebp-1* (PY01365). The basis for this new accessibility is obscure, but it is possible that these proteins form complexes either with each other or other proteins, which could make the antibody binding site cryptic. Our result suggests that in WT parasites there are at least two Py235 erythrocyte binding proteins, Py235EBP-1, (recognized by mAb 25.77 and encoded by PY01365) and Py235EBP-2 (recognized by mAb 25.37 and encoded by PY01185). In the PY01365-KO parasite line, the erythrocyte binding protein is changed to Py235EBP-2 recognized by both mAb 25.77 and 25.37 in the absence of Py235EBP-1. In conclusion, in the absence of Py235EBP-1, invasion of erythrocytes by *P. yoelii* takes place using Py235EBP-2, an alternative Py235 erythrocyte binding protein; modulation of erythrocyte binding appears to occur at the level of the proteins without significant changes in gene expression.

## Materials and Methods

### Ethics statement

All animal work protocols were reviewed and approved by the Ethical Review Panel of the MRC National Institute for Medical Research and approved and licensed by the UK Home Office as governed by law under the Animals (Scientific Procedures) Act 1986 (Project license 80/1832, Malaria parasite-host interactions). Animals were handled in strict accordance with the “Code of Practice Part 1 for the housing and care of animals (21/03/05)” available at http://www.homeoffice.gov.uk/science-research/animal-research/. The numbers of animals used was the minimum consistent with obtaining scientifically valid data. The experimental procedures were designed to minimize the extent and duration of any harm and included predefined clinical and parasitological endpoints to avoid unnecessary suffering.

### Animals and parasites

Female BALB/c mice, with an average weight of 18 to 22 g and 6 to 8 weeks old were obtained from the specific pathogen-free unit at the MRC National Institute for Medical Research. The cloned virulent YM line of P. *yoelii* [46], [51], was obtained from Dr. David Walliker, University of Edinburgh. Parasites were passaged no more than five times in the same mouse strain, before returning to a fresh stabilate.

### Alignment of the py235 gene and protein family sequences

All full-length and partial *py235* gene sequences identified in the database (www.PlasmoDb.org) were retrieved and Clustal X 1.81 [52] was used to align them. Areas of sequence similarity and difference were identified at both the amino acid and nucleotide levels and analysed using Bioedit [53]. The sequence information was then used to design gene-specific reagents, and compare features of the individual sequences.

### Cloning of targeting construct

PY01365 (*py235ebp-1)* gene sequences were amplified from *P. yoelii* YM line genomic DNA using gene specific primers. A 500bp fragment from the 5′UTR of PY01365 (Fragment A) was amplified with forward primer, (restriction sites are underlined), 5′-**gccgg** gggcccACTATAACACTAATTATTTATTATAAAACG-3′ and reverse primer, 5′-**gccgg** aagcttATGTATGTATCTATGTATGCATGCATG-3′. A region from the 3′ coding sequence of PY01365 (Fragment B) was amplified with forward primer, 5′-**gccgg** gaattcACGAACTCACTCGAATACAAAGTCGTTTAG-3′ and reverse primer, 5′-**ggcgg** tctagaATAATTTTTATATTTTGCATCATCATTATTATTATGG-3′. Fragment A PCR product was digested with ApaI and Hind III and Fragment B PCR product was digested with EcoRI and XbaI. The targeting construct was made by the cloning of fragments A and B sequentially into the plasmid vector pBSDHFR, in which the *Toxoplasma gondii* dihydrofolate reductase/thymidylate synthase gene (DHFR/TS) is flanked by the upstream and downstream control elements from *P. berghei* DHFR/TS. First, Fragment A was cloned into pBSDHFR that had been digested with Apa1 and Hind III and the inserted DNA sequence was verified by sequencing. This construct was digested with EcoRI and XbaI, and then Fragment B cloned into it, and its sequence verified. The final targeting construct was digested with the enzymes ApaI and XbaI and inserted by double homologous recombination into the PY01365 gene following transfection of the virulent YM line of *P. yoelii*.

### Transfection of parasites

Transfection of parasites was carried out essentially as described previously [54], [55]. Briefly, erythrocytes containing late stage parasites, were harvested at 20 to 25% parasitaemia and schizonts were purified by centrifugation for 25 min at 600 *g* at room temperature (RT) on a 55% Nycodenz (Nycoprep) cushion (NYCOMED Pharma AS). 5×10^7^ schizonts were mixed with 90 µl AMAXA nucleofactor T-cell solution (plus supplements) and 5 µg of targeting construct DNA was added. These parasites were transfected using AMAXA Nucleofector programme U33. Immediately, 100 µl of RPMI 1640 medium containing 20% foetal calf serum (FCS) was added to the transfected parasites and the suspension injected intravenously (i.v.) into the tail vein of a single mouse. Electroporation of parasites with targeting construct and injection into individual mice was carried out twice independently using the above conditions.

Twenty seven hours post injection, day (D)1, and on D3 and D4 mice were treated with 1 mg kg ^−1^ pyrimethamine, intraperitoneally (i.p.). From day 2 post injection, pyrimethamine was administered continuously in the drinking water at a final concentration of 70 µg/ml. Three sets of control mice were set up. One set was injected with transfected schizonts as above but did not receive any drug treatment, a second set was injected with schizonts in T-cell solution (without DNA or electroporation) and was drug treated as above, and the third set of controls was as the second set but without subsequent drug treatment. The parasitaemia of each set of mice was monitored daily. Stabilates of drug resistant parasites were made and stored in liquid nitrogen, additionally the parasites were used to infect naïve mice (2×10^7^–5×10^7^ parasitized erythrocytes, administered i.v.) for a second round of drug pressure. These mice were given pyrimethamine continuously in their drinking water as above. Parasites were allowed to grow sufficiently for samples to be taken for analysis and for stabilates to be made. After verification of the pyrimethamine-resistant parasites by PCR and Southern blot analysis, the transgenic parasite line was cloned by limiting dilution using 10 mice injected i.v so that each inoculum contained a maximum of one parasite. Four clones were obtained and genotyped.

### Southern blot analysis

Genomic DNA (gDNA) was isolated from leukocyte-depleted, Percoll-purified late stage WT and PY01365-KO parasitized erythrocytes lysed in a buffer containing SDS. The DNA was phenol chloroform extracted and precipitated with ethanol. Various restriction enzymes were used to digest the gDNA and samples were resolved by electrophoresis on a 0.8% agarose gel and transferred onto Hybond N+ in 7.5 mM NaOH overnight. The filter was neutralized in 2 x SSC and UV cross-linked prior to hybridisation [13]. DNA probes used were: Fragment B (see above), and a 739 bp TgDHFR DNA sequence PCR amplified from plasmid DNA using the forward primer: 5′-GCCGGGATCCCATCATTCGACCCTGATATATATAACGA-3′ and the reverse primer: 5′-GCCGGGAATTCATTCTAAAAATTCATAGTAATAAGGTG-3′.

For an estimation of the number of copies of PY01365 in the *P. yoelii* genome, WT gDNA was digested with various restriction enzymes in double digest reactions and the samples transferred onto nylon filters as above. DNA probes used were Fragment B, (as above) and Fragment C derived from the 5′ coding sequence of PY01365, amplified using the forward primer: 5′- ATCATCTGCACCATCATTCGAC-3′ and the reverse primer: 5′- CAATATGGAATCTAATAGACG-3′. DNA probes were labelled using DECAprime II labelling system (Ambion) and hybridized to the filters.

### Pulse field gel electrophoresis

Chromosomes from leukocyte–depleted, Percoll-purified late stage WT and PY01365-KO parasites were fractionated by contour-clamped homogeneous electric field (CHEF) electrophoresis as described [18]. The gel was blotted and hybridized sequentially with three different probes. First a probe that binds to the 5′ coding region of py235ebp-1 (Fragment C); second, a probe that binds to the 3′ coding region of py235ebp-1 (Fragment B); and finally, a probe that binds to the 3′ UTR of DHFR/TS.

### Protein purification, mass spectrometry analysis and bioinformatics

Erythrocytes were harvested from BALB/c mice infected with *P. yoelii* YM WT or PY01365-KO lines and depleted of leukocytes [23]. Py235 protein from both supernatant and detergent solubilized parasite preparations was purified on separate columns by affinity chromatography using mAb 25.77 as described previously [20]. Affinity chromatography using mAb 25.37 was also carried out as above but only using WT parasites.

Proteins eluted from the affinity columns were subjected to SDS-PAGE under reducing conditions on a 5% polyacrylamide gel and visualised using colloidal blue stain (Novex). Bands were excised, reduced, alkylated and digested with trypsin [56]. Peptide mass fingerprinting was carried out using a Reflex III MALDI-ToF mass spectrometer (Bruker Daltonik, Germany). The peptide mass fingerprints were used to query sequences in both the rodent malaria database [15] and the general non-redundant database at the National Centre for Biotechnology Information, (NCBI; http://www.ncbi.nlm.nih.gov). The gene accession numbers identified were used to carry out further searches of the NCBI database to obtain full gene sequence information.

### PCR amplification of a py235 gene to link two contigs

Two of the three py235 sequences identified by mass spectrometry analysis using peptides from 25.37-affinity purified protein, did not correspond to full-length genes, instead they corresponded to two contigs, PY05995 and PY03534. A gene specific forward primer was designed based on DNA sequences from the last 200 nucleotides of the 3′ coding sequence of PY05995 (Forward primer 5′-GAAATGAAACGTACAAAAGATGACATC-3′), and a reverse primer was designed based on the first 200 nucleotides of the 5′ coding sequence of PY03534 (Reverse primer 5′-CTGTATATGATTGTTCTATTAAATTAC-3′). Using WT gDNA, PCR amplification was carried out using Pfu ultra DNA polymerase (Strategen). The PCR product was directly sequenced (Cogenics), analysed, aligned and assembled with the PY05995 and PY03534 contigs to create a single py235 gene, using Bioedit [53].

### Quantitative real time RT-PCR (qPCR)

Total RNA was prepared [57] from leukocyte-depleted, Percoll-purified late stage WT and PY01365-KO parasitized erythrocytes using Trizol (Invitrogen Life Technologies). RNA samples were first treated with RNase-Free DNase I (Quiagen) and cleaned up using RNeasy MiniElute to remove contaminating gDNA. First strand cDNA was synthesized using 1 µg RNA, AMV reverse transcriptase (RT) and random primers according to the manufacturer's instructions (Promega). RT-PCR amplification using the synthesized cDNA was carried out and samples amplified without the addition of RT were included as controls.

Gene specific primers were designed for the Py235 genes of interest PY01365, PY01885, and PY05995/PY03534 and the reference genes, *P. yoelii* erythrocyte binding protein (PyEBL) and Pyβ-tubulin (Table S1). Short regions of the genes (150bp–193bp) were amplified using gDNA extracted from purified late stage WT *P. yoelii* YM parasites. cDNA was used as a template to PCR amplify Pyβ-tubulin. PCR products were cloned into TA vector, and clones containing inserts were identified by PCR and the insert DNA verified by sequencing.

qPCR was carried according to the MIQE guidelines [58]. qPCR reactions (25 µl) were set up in triplicates in Absolute SYBR Green mix (containing Thermo-Start, DNA polymerase and ROX Dye) (Abgene), 0.2 µM each primer and 1 µl cDNA and amplified in an ABI Prism 7000 Sequence Detection System (Applied Biosystems). Cycle conditions were 50°C, 2 min; 95°C, 15 min; 40 cycles of 95°C, 15 s; 60°C, 1 min. gDNA was used to check that amplification efficiencies of primers were comparable and plasmids used to generate standard curves were included in each assay. Transcript levels for each gene in the WT and PY01365-KO parasite lines were quantified and normalized with Pyβ-tubulin and PyEBL. For analysis, cDNA prepared from two independent RNA samples was used.

### RNA-Seq analysis

Parasites were purified using a MACS type-D depletion column with a SuperMACS II magnetic separator (Miltenyi Biotec GmbH) [59]. RNA from the purified WT and PY01365-KO parasite lines, prepared as described above, was sequenced and analyzed as described [44]. Briefly, the RNA of both samples was depleted of ribosomal RNAs with exonuclease and sequenced on an Illumina GA II platform using the Illumina RNA-seq protocol. Of the approximately 61 million 76-base pair paired-end reads per run, around 95% percent mapped with SSAHA2 [60] against the *P. yoelii* 17XNL genome sequence (GeneDB: ftp: ftp://ftp.sanger.ac.uk/pub/pathogens/P_yoelii/June_2010/). From the coverage of the uniquely mapped Illumina reads, a perl script was used to calculate the geometric mean for expression of each predicted gene, representing the level of messenger RNA. We compared the expression in both samples by obtaining the ratio of expression values for each gene.

### Immunofluorescent antibody (IFA) assay

IFA assay of Py235 protein expression in WT and PY01365-KO parasites was carried out using mAb 25.77 on formaldehyde fixed parasitized erythrocytes, followed by Alexa Fluor 488-conjugated affinity purified goat anti-mouse IgG (Molecular Probes). In colocalization studies EBL was detected using rabbit antibodies provided by Dr Osamu Kaneko. Alternatively the slide was first probed with mAbs specific for either RON4 (48F8) or for AMA1 (45B1) [39] followed by Alexa Fluor594 congugated secondary antibody. After washing, this was followed by incubation with mAb 25.77 directly congugated to Alexa Fluor488. In a separate assay, Alexa Fluor 488-conjugated 25.77 mAb was used to probe thin blood smears of mixed stage WT *P. yoelii* YM parasites, followed by Alexa Fluor 594-conjugated 25.37 mAb in a dual labeling experiment [61]. mAbs were labeled with Alexa Fluor (Molecular Probes) succinimidyl esters according to the manufacturer's instructions. The labeled antibodies were separated from excess labelling reagent by gel filtration on PD-10 columns (Amersham Pharmacia), eluted using PBS/1% BSA. All slides were examined and images captured on an Axioplan 2 imaging system (Zeiss).

### Western blotting

Percoll-purified late stage parasites were solubilized under reducing conditions in a buffer containing DTT, resolved by SDS-PAGE on a 5% Bis-Tris polyacrylamide gel and transferred onto nitrocellulose membrane. Primary antibodies (at 10 µg/ml) were used to immunostain the membrane and were detected by incubation with HRP-congugated goat anti-mouse IgG (H+ L) antibody (Bio-Rad) and the ECL Western Blotting detection reagent (GE Healthcare/Amersham). Protein bands were visualized on a Kodak BioMax MR film. The blots were stripped with Restore PLUS according to the manufacturer's instructions and then probed with mAb 48F8 to detect PyRON4 [41] as a control for protein loading.

### Erythrocyte binding assay and immunoprecipitation

[^35^S]methionine/cysteine (Promix, GE Healthcare, Little Chalfont, UK)) radiolabeled proteins from *P. yoelii* YM (WT and PY01365-KO) either released into culture supernatant, extracted in a buffer containing 0.5% (w/v) sodium deoxycholate, or eluted from erythrocytes were immunoprecipitated using mAbs 25.77 and 25.37, hyperimmune serum (HIS) and normal mouse serum (NMS). The erythrocyte binding assay and immunoprecipitations were carried out as described previously [24].

### Course of parasite infection in vivo

Groups of 5 Balb/c mice were infected i.v. on day 0 with either 200, 1000, or 5000 WT or PY01365-KO parasitized erythrocytes [62]. Blood smears from each mouse were made daily from D3, stained with Giemsa's reagent and infected cells counted to monitor the course of infection.

## Supporting Information

Figure S1 Western blot analysis of lysates from WT and PY01365-KO parasites. Percoll purified late stage parasites of both PY01365-KO (Lanes 1) and WT (Lanes 2) lines were solubilized under reducing conditions, resolved by SDS-PAGE on a 5% polyacrylamide gel and transferred onto nitrocellulose membrane. The membrane was probed with either (A) mAb 25.77, or (B) mAb 25.37 and all the tracks were then probed with mAb 48F8 (PyRON4) as a loading control. The positions of the proteins recognized by the mAbs are indicated.
(1.84 MB TIF)

Figure S2 Scatter plot of log expression measured with the geometric mean, comparing all genes of the WT and the KO parasite line.
(0.38 MB TIF)

Table S1 Unique primers used in quantitative real time RT-PCR (qPCR) amplification.
(0.06 MB DOC)
