# Supplementary figures and images for: Targeted Disruption of py235ebp-1: Invasion of Erythrocytes by Plasmodium yoelii Using an Alternative Py235 Erythrocyte Binding Protein
Source: PLoS Pathog. 2011 Feb 17;7(2):e1001288. doi: 10.1371/journal.ppat.1001288 (PMC3040676; doi:10.1371/journal.ppat.1001288)

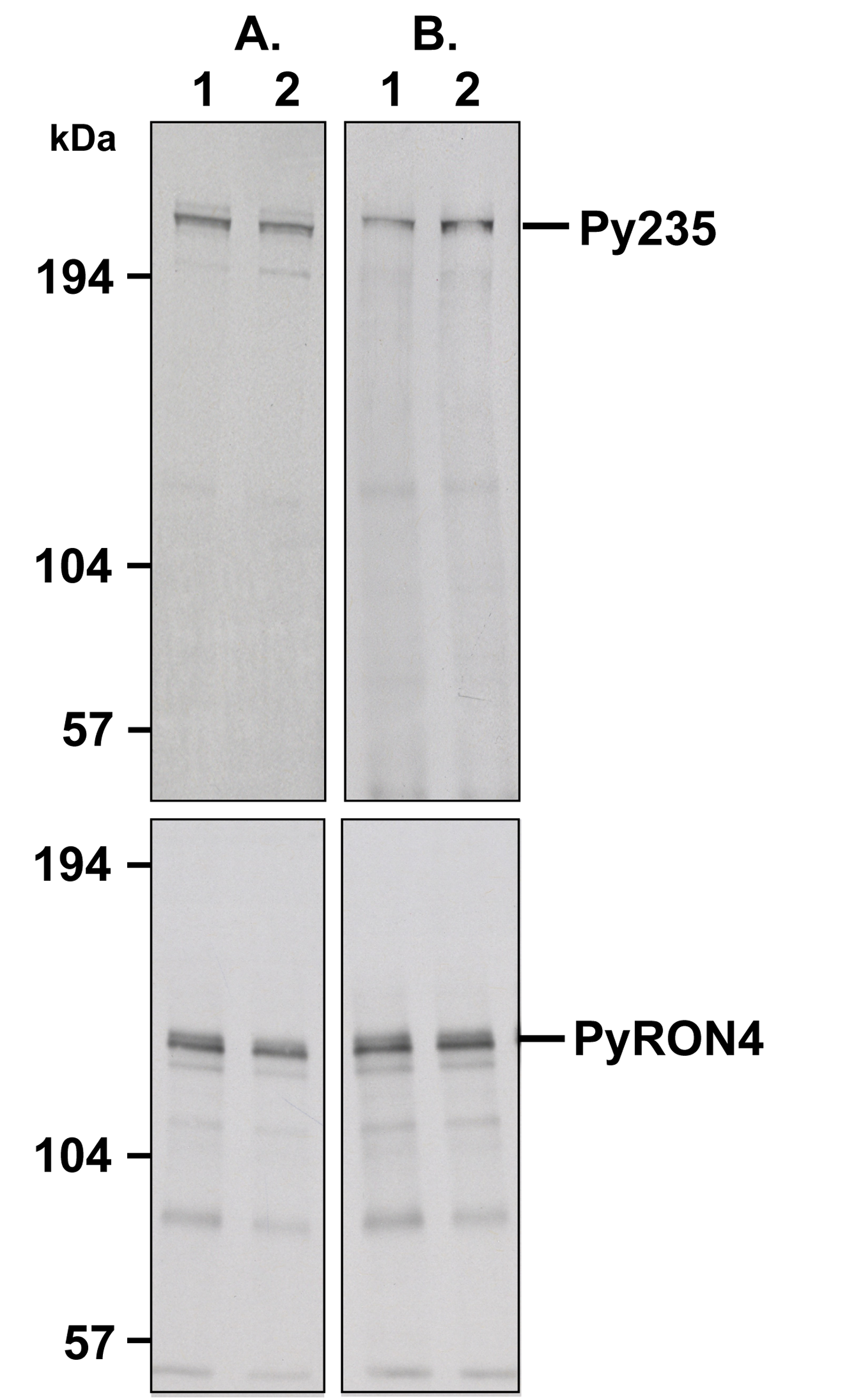

Supplement: Figure S1 — Western blot analysis of lysates from WT and PY01365-KO parasites. Percoll purified late stage parasites of both PY01365-KO (Lanes 1) and WT (Lanes 2) lines were solubilized under reducing conditions, resolved by SDS-PAGE on a 5% polyacrylamide gel and transferred onto nitrocellulose membrane. The membrane was probed with either (A) mAb 25.77, or (B) mAb 25.37 and all the tracks were then probed with mAb 48F8 (PyRON4) as a loading control. The positions of the proteins recognized by the mAbs are indicated. (1.84 MB TIF) [file ppat.1001288.s001.tif]

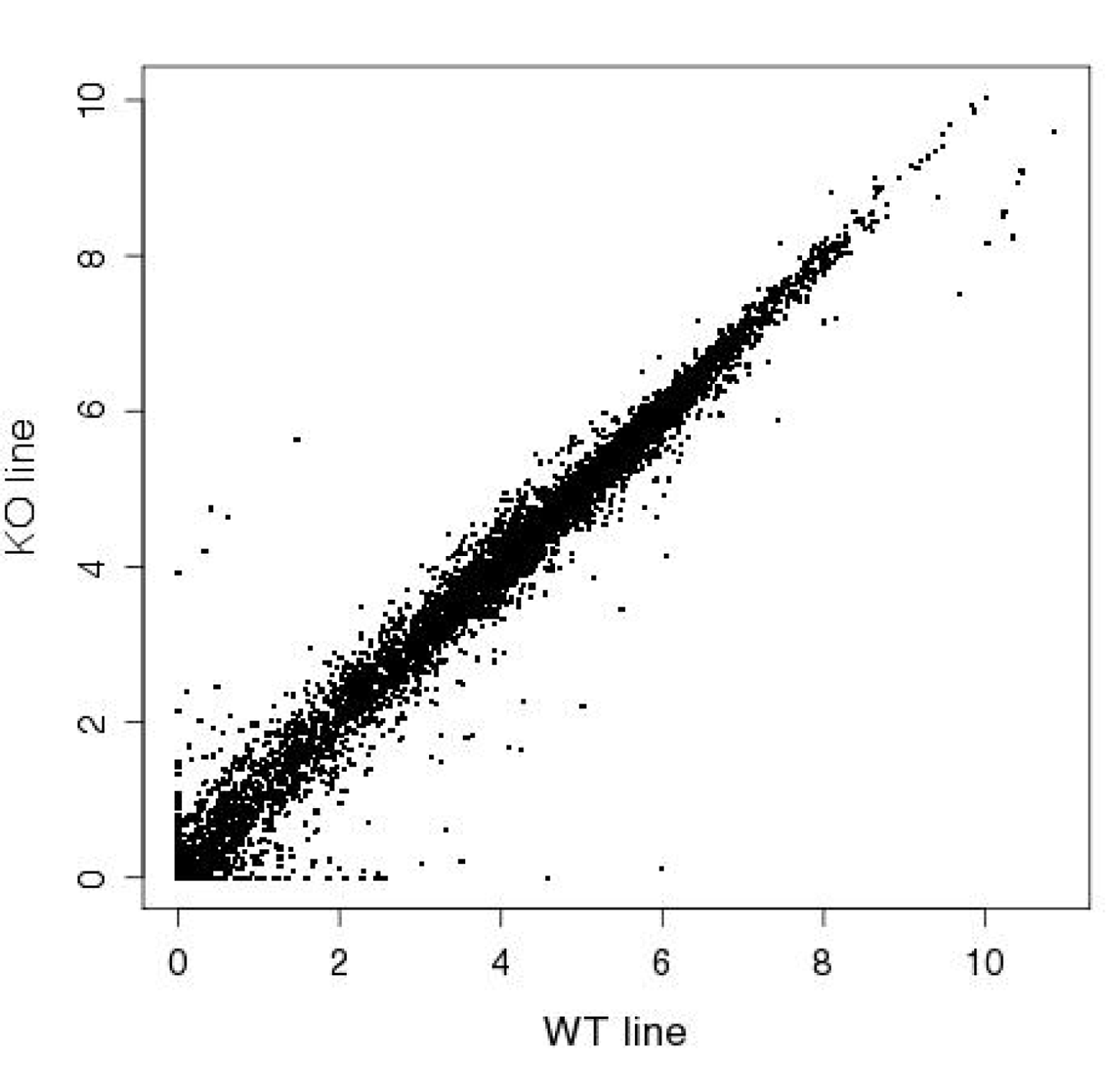

Supplement: Figure S2 — Scatter plot of log expression measured with the geometric mean, comparing all genes of the WT and the KO parasite line. (0.38 MB TIF) [file ppat.1001288.s002.tif]
