# Supplementary material for: Targeted Disruption of py235ebp-1: Invasion of Erythrocytes by Plasmodium yoelii Using an Alternative Py235 Erythrocyte Binding Protein
Source: PLoS Pathog. 2011 Feb 17;7(2):e1001288. doi: 10.1371/journal.ppat.1001288 (PMC3040676; doi:10.1371/journal.ppat.1001288)
Supplement: Table S1 — Unique primers used in quantitative real time RT-PCR (qPCR) amplification. (0.06 MB DOC) [file ppat.1001288.s003.doc]

# **SUPPLEMENTARY INFORMATION**

**Table S1.** Unique primers used in quantitative real time RT-PCR (qPCR) amplification

| Gene | Primer sequence 5’ to 3’ | Location of amplified region |
| --- | --- | --- |
| PY01365F | ATCATCTGCACCATCATTCGAC | 399-576 |
| PY01365R | CAATATGGAATCTAATAGACG |  |
| PY01185F | GCATATTCCCAATTTTACTATGCG | 700-893 |
| PY01185R | ATTTCCATTTGAAATACCATATAA |  |
| PY05995/03534F | GAAGATTCCAAATCGCAATATGTG | 718-911 |
| PY05995/03534R | GTTCCCAATTGAAAGATCATATGA |  |
| *PY04764F | GTTAATGAATGTAAGGAAAAA | 346-502 |
| PY04764R | CTTTCGTTTTATTATCCGGTA |  |
| **PY05711F | AGCAGGCCAATGTGGTAATC | 24-237 |
| PY05711R | ACCTGCACGAACACTATCCA |  |

*PY04764 is PyEBL

**PY0571 is Py β-tubulin
